# “Register and Roll”: A Novel Initiative to Improve First Door-to-Balloon Time in ST Elevation Myocardial Infarction

**DOI:** 10.1155/2012/616940

**Published:** 2012-10-08

**Authors:** Sachin Kumar Amruthlal Jain, Yousif Ismail, Michael Shaw, Shukri David, Patrick Alexander

**Affiliations:** ^1^Division of Cardiology, Providence Heart Institute, 16001 West Nine Mile Road, Southfield, MI 48075, USA; ^2^Division of Internal Medicine, Providence Hospital, Southfield, MI 48075, USA; ^3^Department of Patient Care Research, Providence Hospital, Southfield, MI 48075, USA

## Abstract

*Objective*. We examined the cause of transfer delay in patients with an acute ST-segment myocardial infarction (STEMI) from non percutaneous coronary intervention (PCI) capable to PCI capable hospitals. We then implemented a novel, simple, and reliable initiative to improve the transfer process. *Background*. Guidelines established by the ACC/AHA call for door-to-balloon times of ≤90 minutes for patients with STEMI. When hospital transfer is necessary, this is only met in 8.6% of cases. *Methods*. All patients presenting with STEMI to a non-PCI capable hospital from April 2006 to February 2009 were analyzed retrospectively. After identifying causes of transfer delay the “Register and Roll” initiative was developed. An analysis of effect was conducted from March 2009 to July 2011. *Results*. 144 patients were included, 74 pre-initiative and 70 post- initiative. Time to EMS activation was a major delay in patient transfer. After implementation, the EMS activation time has significantly decreased and time to reperfusion approaches recommended goal (Median 114 min versus 90 min, *P* < 0.001), with 55% in <90 minutes. *Conclusion*. “Register and Roll” streamlines the triage process and improves hospital transfer times. This initiative is easily instituted and reliable in a community hospital setting where resources are limited.

## 1. Introduction

More than 600,000 patients present at the emergency department with previously undiagnosed ST segment elevation myocardial infarction (STEMI) and an additional 300,000 with recurrent myocardial infarction yearly [1]. The goal of reperfusion therapy for acute STEMI is to reestablish blood flow to the affected coronary artery as soon as possible. Rapid time to treatment with primary percutaneous coronary intervention (PPCI) is associated with lower mortality rates compared with fibrinolytic therapy with each 30 minutes of delay increasing the relative risk of mortality at one year by 7.5%. Previous studies have demonstrated marked survival advantage in patients for whom PPCI is performed to achieve timely reperfusion of the infarct-related artery [2, 3]. National guidelines developed by the American College of Cardiology/American Heart Association (ACC/AHA) state that hospitals treating STEMI patients with percutaneous coronary intervention (PCI) should strive to achieve a median door-to-balloon (D2B) time of less than 90 minutes [4]. Recently, technological advances along with collaborative work with EMS providers have facilitated treatment, however, not all communities have access, funding, or the training to achieve the standards set by the ACC/AHA. Despite these guidelines and successful efforts to reduce D2B times [5, 6], there remains a persistent challenge in achieving goal D2B times when patients first present to non-PCI-capable hospitals and require transfer to a PCI-capable hospital for reperfusion. In a recent analysis of the National Cardiovascular Data Registry, only 8.6% of STEMI patients transferred for PPCI reported with D2B times of ≤90 minutes [7]. We term the time from initial presentation at the non-PCI capable hospital to first balloon inflation time, the 1st door-to-balloon time (1stD2B). Our study aimed to evaluate key time intervals in the 1st D2B time of patients transferred to our hospital for PPCI with the goal of identifying potential causes for delay in those with prolonged 1st D2B times, followed by an initiative to reduce delays based on our initial findings.

## 2. Materials and Methods

The study's design was approved by the Providence Hospital Institutional Review Board (IRB) prior to data collection. We conducted a retrospective analysis of all patients undergoing PPCI at Providence Hospital's Heart Institute for acute STEMI from April 2006 to December 2008 who were transferred from the non-PCI capable Providence Park Hospital. Providence Hospital is a 365-bedded community, non-for-profit hospital located in Southfield, serving metropolitan Detroit and its suburbs. Providence Park Hospital is a sister hospital which is 200 bedded located in Novi, approximately 18 miles away from the Providence Hospital. Available paper and electronic charts as well as diagnostic reports were reviewed from both participating hospitals. Measured outcomes included left ventricular ejection fraction (EF) at discharge, cardiogenic shock on presentation, and length of stay (LOS). Patients under 18 years of age and those with initial presentation other than chest pain or an indeterminate initial electrocardiogram (ECG) were excluded. 

Analysis of the key time intervals was performed in all patients. The time to ECG, ECG to EMS call, time to EMS arrival, total time to transport (door-in-door-out time), en route transportation time, and time to balloon inflation were recorded. The 1st D2B was reported in all patients and encompassed the time from initial presentation to balloon inflation in the catheterization laboratory. 

The register and roll initiative was implanted to facilitate transfer from the presenting emergency room. Two years after implementation of the register and roll initiative, we analyzed key points in triage for comparison. 

Statistical Analysis was performed using SPSS software version 15.0. Continuous variables were analyzed using a two sample *t*-test or a Mann-Whitney test when medians were expected to be divergent. Categorical values were addressed using a Chi-square test or Fisher's Exact test. 

## 3. Register and Roll Initiative

 The overall effort was to begin transfer with a minimum number of procedures performed in the ER. This process streamlined the triage of patients at the primary hospital. It involved simple guideline, a Cath Kit and prompt EMS notification. Patient's presenting with a STEMI received a single intravenous line, anticoagulation via single bolus, antiplatelet treatment (aspirin plus clopidogrel or prasugrel), and a single blood draw for basic labs. Catheterization lab compatible IV locks and ECG tabs were used. Early EMS notification was prioritized along with a rapid consultation with an interventional cardiologist is initiated.

## 4. Results

A total of 144 patients were included in the analysis. The pre-register and roll group consisted of 74 patients transferred from Providence Park Hospital, a distance of 18 miles. In post-initiative, we analyzed data on 70 patients. A comparison of baseline characteristics between the two groups revealed a slightly younger preinitiative population, but with similar comorbidity prevalence (Table 1). As expected, mean time to reperfusion (1st D2B) was significantly prolonged 130 versus 93 minutes (*P* < 0.001) in the pre-versus postgroups, respectively. 

**Figure 1:**
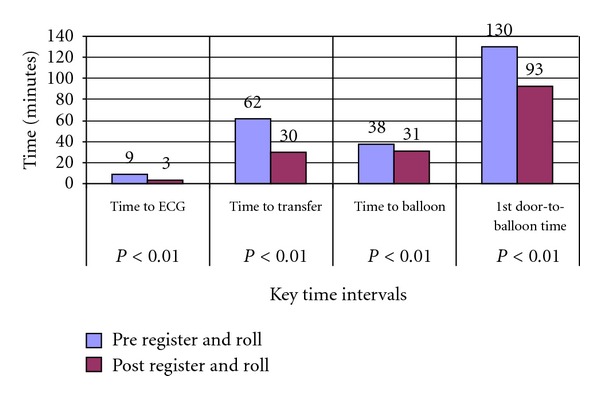
Key time intervals of pre- and postregister and roll initiative (mean time intervals).

**Figure 2:**
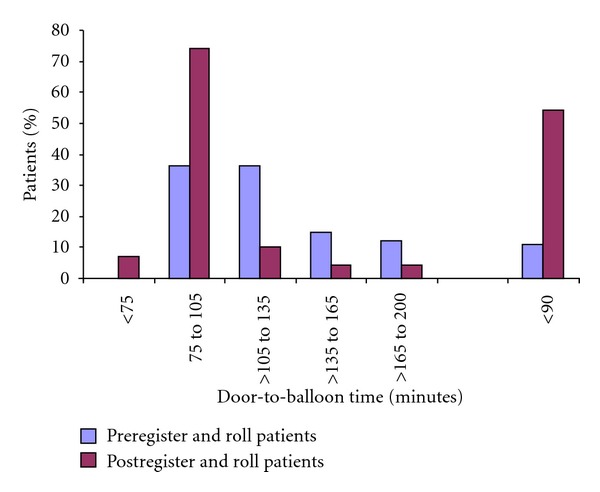
Percent patients with door-to-balloon times.

Analysis of the key time intervals in the study group demonstrates the dramatic effect our initiative had in reducing triage times. The documented time to ECG was reduced threefold compared to the control group (5 versus 1.5 minutes, *P* < 0.001Table 2). Time to EMS activation was reduced significantly, resulting in reduced time to transfer times. Time to transfer was not affected by changes in EMS procedures as EMS arrived in less than 10 minutes for both groups and as expected, both groups had similar transport times. Time to Transfer included the time patient arrived to the first hospital and left the first hospital (door-in-door-out time). Changes to procedures at the non-PCI hospital also reduced clinical times in the catheterization laboratory as evidenced by a 5-minute reduction in door-to-balloon times (Table 2). The mean key time intervals are shown in graphical representation in Figure 1. Overall, our 1st D2B times were reduced by the register and roll initiative by nearly 72% overall. More significantly, implementation resulted in achieving a 1st D2B time of 90 minutes in the majority of patients (Figure 2). 

Although our study was not powered for outcomes, we did access length of stay and discharge ejection fraction (EF) in the two groups. We found a significantly higher EF (47.3% versus 53.4%, *P* = 0.02) in the postregister and roll group, while there were a similar number of MACE events in each group (Table 3).

## 5. Discussion

As illustrated in prior studies, transfer of STEMI patients for PPCI has been associated with significantly longer door-to-balloon times compared to those presenting directly to a PCI center [7, 8]. Our preinitiative data showed similar delays in reperfusion with a “first” door-to-balloon (1st D2B) times of nearly 1 and a half hours. The 1st D2B time in the majority of patients was 1.5 to 3 hours, with only 7.8% achieving the national set goal of ≤90 min (Figure 2). This is similar to the National Cardiovascular Data Registry analysis (2005 to 2006) involving over 15,000 patients and 491 hospitals where the total door-to-balloon time of ≤90 minute was achieved in only 8.6% of patients [7]. 

Delays in the timely request for EMS and an overall prolonged total time-to-transport, (door-in-door-out time), contributed to the significantly prolonged 1st D2B times seen in the STEMI patients transferred to our hospital for PPCI. Analysis of key time periods reveals an appropriated EMS response time (median = 7 to 8 minutes) with acceptable median transportation times of 25 to 30 minutes between the presenting institution to our hospital. Furthermore, the initiation of medications at the presenting hospital such as intravenous (IV) boluses and drips, as well as extensive lab request may have contributed to the prolonged door-in-door-out times. Prior to implementation of register and roll, upon arrival for PPCI, the median time to balloon inflation was 34 minutes which represented a mild delay that was attributed to the time taken to correct and/or change noncompatible IV lines and drips between the transferring hospital, EMS, and our hospital. Furthermore, several patients presented with inappropriate gowns and/or translucent ECG leads requiring correction, which posed further challenges in reducing the total door-to-balloon times. When compatible materials were used in each hospital, the time to balloon was significantly reduced. 

Possibly due to the significantly longer 1st D2B times in those transferred for PCI prior to the changes, there was a noted difference in the EF at discharge between groups in our study. However, we noted no difference in outcomes between the groups. The lack of difference in outcomes in our study may be related to a younger age of the pre-initiative group in addition to a relatively small sample size.

Innovative strategies to meet the set guidelines and further reduce door-to-balloon times have been described in the literature and include activation of the catheterization lab by EMS personnel, emergency room physicians, single call to a central paging operator, prehospital activation of the catheterization lab, prompt response of ≤20 minutes by cath lab personnel, and 24-7 on-site interventional cardiologist [5, 8, 9]. More recently, the use of pre-hospital ECG transmission to the emergency room and subsequent activation of the catheterization lab were effective in reducing both the door-to-needle times as well as the D2B times in a review of nearly 2,000 patients in the ACTION Registry [10]. Additionally, utilization of pre-hospital ECG was also shown to decrease the D2B during daytime as well as off hours which is an ongoing concern regarding the overall availability of patient care during the night hours and weekends [11, 12]. 

In accordance with the ACC/AHA Mission: Lifeline, the challenges to reduce the D2B times in STEMI patients persist among all hospitals especially in those whom transfer of STEMI patients is required. The use of pre-hospital ECG by EMS personnel and establishment of STEMI receiving networks (SRC) especially targeted to the effective transferring of STEMI patients to PCI capable hospitals have been recently reported [13]. Continuous improvement and quality assessment between PCI centers and neighboring hospitals is necessary if we are to continue to improve the care of patients with STEMI. Perhaps a “register and roll” approach is needed to rapidly triage and transfer those patients who present to hospitals not capable of performing primary PCI for STEMI. This would entail the current foundations stated in the D2B Alliance [5] in addition to avoidance of IV drips when oral medication can be administered, an emergency room focused examination with limited lab draws, use of universal gowns and equipment (IV tubing, ECG leads, etc.) between local hospitals, and perhaps a 24-7 on-site dedicated STEMI ambulance with a transfer team for prompt mobilization, that is, reducing the door-in-door-out time and subsequently the 1st D2B time. 

## 6. Conclusion

Despite ongoing reductions in the D2B in STEMI patients presenting directly PPCI, there remain considerable delays in those presenting to non-PCI-capable hospitals. Potential improvements center on decreasing the door-in-door-out times by standardizing equipment between participating hospitals along with rapid transport to the PCI center (either rapid contact of EMS services or use of an onsite hospital designated ambulance to rapidly transport patients to a PCI center). Furthermore, adherence to the D2B Alliance strategies along with continuous quality assessment and improvements between participating hospitals, emergency departments, and EMS is need to improve the transfer of patients with STEMI. 

## Figures and Tables

**Table 1:** Demographics of pre- and postinitiative patients.

Demographic	Pre (*n* = 74)	Post (*n* = 70)	Significance
Male gender (% male)	88	69	*P* = 0.3
Age (mean ± SD)	56 ± 14	62 ± 12	*P* = 0.007
Hypertension	54.1%	64.3%	*P* = 0.6
Diabetes	18.9%	24.3%	*P* = 0.6
Dyslipidemia	59.85%	77.1%	*P* = 0.4
Tobacco abuse	60.8%	51.4%	*P* = 0.6

**Table 2:** Reduction of time intervals by the register and roll initiative (median time intervals).

Outcome (in minutes)	Pre (*n* = 74)	Post (*n* = 70)	Significance
Median time to ECG (interquartile range)	5 (3–9)	1.5 (1–4)	*P* < 0.01
Median time ECG to transfer (IQR)	44 (34–57)	24 (25–41)	*P* < 0.01
Median time EMS activation to arrival (IQR)	8 (5–10)	7 (4–9)	*P* < 0.10
Median time to transfer (IQR)	51 (39–70)	26.5 (17–36)	*P* < 0.01
Median transport time (IQR)	26 (21–30)	30 (25–41)	*P* = 0.09
Median time to balloon (IQR)	34 (27–44)	29 (23–36)	*P* < 0.01
Median 1st door-to-Balloon time (IQR)	114 (102–139)	89.5 (83–100)	*P* < 0.01

Notes: Time ECG to transfer included EMS activation after first diagnostic EKG, EMS arrival, and the patient leaving the primary hospital. Time to transfer included the patient's arrival to the first hospital and leaving the first hospital (door-in-door-out time).

**Table 3:** Outcomes in the pre- and postinitiative patient populations. MACE is major adverse cardiac events (myocardial infarction, unstable angina, cardiovascular death, revascularization, fatal/nonfatal cerebrovascular accident, peripheral arteriopathy, aortic event), EF: ejection fraction measured at discharge.

Outcome	Pre (*n* = 74)	Post (*n* = 70)	Significance
Length of stay	3.1 ± 2.0	3.0 ± 2.0	*P* = 0.28
EF (% ± SD)	47.3 ± 0.1	53.4 ± 12.0	*P* = 0.02
MACE	11	9	*P* = 0.91
